# Coherent Spin Waves in Curved Ferromagnetic Nanocaps of a 3D‐Printed Magnonic Crystal

**DOI:** 10.1002/smll.202508983

**Published:** 2025-12-17

**Authors:** Huixin Guo, Kilian Lenz, Mateusz Gołębiewski, Ryszard Narkowicz, Jürgen Lindner, Maciej Krawczyk, Dirk Grundler

**Affiliations:** ^1^ School of Engineering Institute of Materials, Laboratory of Nanoscale Magnetic Materials and Magnonics École Polytechnique Fédérale de Lausanne (EPFL) 1015 Lausanne Switzerland; ^2^ Institute of Ion Beam Physics and Materials Research Helmholtz‐Zentrum Dresden–Rossendorf Bautzner Landstr. 400 01328 Dresden Germany; ^3^ Institute of Spintronics and Quantum Information Faculty of Physics and Astronomy Adam Mickiewicz University Uniwersytetu Poznańskiego 2 61‐614 Poznań Poland; ^4^ School of Engineering Institute of Electrical and Micro Engineering École Polytechnique Fédérale de Lausanne (EPFL) 1015 Lausanne Switzerland

**Keywords:** 3D magnonics crystals, ferromagnetic resonance, ferromagnetism, magnonics, micromagnetism, nanostructures

## Abstract

Coherent magnon modes in a truly 3D magnonic crystal have yet to be investigated. This scientific gap exists despite numerous theoretical predictions of miniband formation and edge modes with topological protection. Such properties are key to advancing nanomagnonics for ultrafast data processing. In this work, a scalable nanotechnology for fabricating 3D magnonic crystals embedded in an on‐chip microresonator is presented. It is realized by two‐photon lithography of a 3D woodpile structure and atomic layer deposition of 30‐nm‐thick nickel film. Operated near 14 and 24 GHz, the microresonator output revealed numerous coherent magnons with distinct angular dependencies reflecting the underlying face‐centered cubic lattice. Micromagnetic simulations show that the edge modes are localized within curved nanocaps and remain robust against changes in field orientation. Along an edge, they exhibit an unexpected phase evolution. These findings advance the development of functional microwave circuits with 3D magnonic crystals and strengthen their visionary prospects for edge‐dominated magnon modes.

## Graphical abstract

A nanoprinted 3D magnonic woodpile crystal is measured by microresonator ferromagnetic resonance. Micromagnetic simulations are performed to analyze and visualize the detected coherent spin‐wave modes along the nickel‐coated tubes and caps for various field angles. The woodpile exhibits an unexpected phase evolution and robust edge modes.

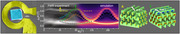

## 1 Introduction

3D nanomagnetic systems have gained significant attention in recent years, serving as platforms for both fundamental discoveries and practical applications.^[^ ^1^, ^2^, ^3^, ^4^, ^5^, ^6^, ^7^, ^8^ ^]^ Their geometry and topology in 3D architectures give rise to new physics.^[^ ^6^, ^9^, ^10^, ^11^, ^12^, ^13^ ^]^ Meanwhile, their inherent advantages—high storage density, device miniaturization, and numerous interconnections—make them promising candidates for next‐generation applications, including memory, logic, and neuromorphic computing. Among these, 3D magnonic crystals stand out as systems that utilize spin waves instead of charge transport, thereby eliminating Joule heating and making them highly attractive for energy‐efficient information processing. Additionally, they offer reprogrammable magnonic band structures, enabling tunable spin‐wave propagation for advanced wave‐based computing,^[^ ^1^, ^14^ ^]^ including topologically protected edge magnon modes in 3D architectured magnetic materials.^[^ ^15^ ^]^

Although the concept of 3D racetrack memory was first introduced in 2008,^[^ ^16^ ^]^ research on 3D magnetic nanodevices has remained mostly theoretical due to fabrication challenges. Only recently have advances in nanofabrication techniques, such as focused electron beam‐induced deposition (FEBID),^[^ ^17^ ^]^ nanosphere‐lithography,^[^ ^18^, ^19^, ^20^ ^]^ two‐photon lithography (TPL),^[^ ^21^, ^22^, ^23^, ^24^, ^25^, ^26^ ^]^ and block copolymer templating,^[^ ^27^ ^]^ have enabled the fabrication of 3D magnetic nanostructures. These developments have now made possible direct experimental investigations, including spin‐wave characterization and magnetic imaging.^[^ ^10^ ^]^ Although the racetrack has recently been freed from the substrate, a scalable nanotechnology is not yet available for its complete 3D implementation.^[^ ^28^ ^]^

Despite recent advances, a systematic study of spin dynamics in 3D magnonic crystals remains lacking. Previous investigations of thermally or coherently excited spin waves in 3D magnetic systems have not exhibited full 3D periodicity. For instance, in Ref. [29], FEBID‐fabricated, 40‐nm‐thick Co–Fe nanovolcano structures were studied using spin‐wave resonance spectroscopy, revealing that the ring‐shaped regions support high‐frequency eigenmodes, whereas the crater regions host lower‐frequency excitations. Sahoo et al.^[^ ^30^ ^]^ investigated coherent spin‐wave modes in a 3D artificial spin‐ice lattice using Brillouin light scattering (BLS), with simulations predicting both localized and extended modes. Still, the system consisted of only four magnetic sublayers. The absence of several periods along the vertical direction precludes the classification of the previously investigated samples as 3D magnonic crystals. More recently, gyroid‐based ferromagnetic nanostructures have been explored both experimentally and numerically for their collective spin‐wave dynamics.^[^ ^31^ ^]^ However, due to the presence of multiple crystallographic domains in large‐volume samples, the measured spectra only provided an averaged response.

To address these limitations, the authors of Ref. [32] developed a scalable technology for creating true 3D magnonic crystals by combining TPL with atomic layer deposition (ALD) of ferromagnetic nickel (Ni). Using micro‐focused BLS and exploring incoherent magnons excited in 3D ferromagnetic woodpile structures by thermal fluctuations, they identified surface modes at ultrahigh frequencies, with shifts of up to 10 GHz between surface and bulk modes. As BLS is inherently surface‐sensitive, the magnon modes deep within the bulk of the samples and on their side facets remained inaccessible. The integration of 3D magnonic crystals into on‐chip microwave devices, as well as their coherent spin‐wave excitation, has yet to be demonstrated.

## 2 Results

In this work, we report an essential advancement toward the development of functional 3D magnonic devices by integrating a full‐fledged 3D magnonic crystal into a microwave resonator. Its thin‐film micro‐loop antenna is operated at approximately 14 and 24 GHz. The comparison between the experimental spectra and micromagnetic simulations reveals that the high sensitivity of the microresonator enables the detection of both bulk and cap‐localized modes. The analysis uncovers several classes of spin‐wave excitations, including extended modes propagating along the principal axes of the nanotubes, as well as spatially confined modes localized in specific structural regions, such as the conformally coated end‐caps of the woodpile lattice. For some of these localized modes their eigenfrequencies are found to be strikingly independent of the field orientation over a wide range of field strengths and angles. These results represent a significant advance toward the coherent excitation and control of spin waves in 3D magnonic crystals. Furthermore, they establish a viable route for integrating 3D nanomagnetic architectures into functional microwave circuits through inductive coupling, highlighting their potential for reconfigurable, miniaturized, and energy‐efficient magnonic devices.

### 2.1 Nanofabrication and Magnetostatic Simulations

We fabricated the magnonic crystals using the scalable additive manufacturing process, combining TPL of a polymeric nanotemplate with Ni‐ALD as described in the Experimental Methods Section and Refs. [32, 33]. Using this 3D‐printing methodology, we fabricated micrometer‐high woodpiles consisting of 12 × 12 × 6 unit cells of ferromagnetic nanotubes arranged in a face‐centered cubic (fcc) lattice. This polymeric nanotemplate was conformally coated by ALD with a 5‐nm‐thick Al_2_O_3_ layer, serving as an insulating and adhesion‐promoting interface, followed by a 30‐nm‐thick Ni layer deposited from nickelocene as the metal‐organic precursor.

**Figure 1:**
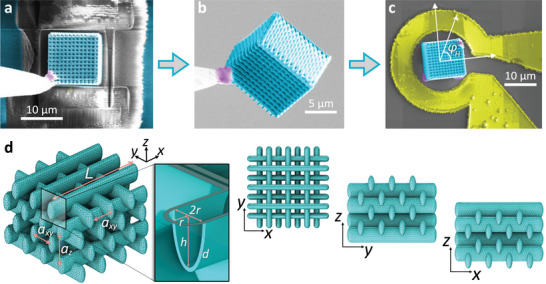
a), False‐colored scanning electron microscopy (SEM) image of the Ni‐coated (blue) woodpile structure on the Si substrate (gray). b) Released woodpile structure attached to the micromanipulator. Platinum (Pt, purple) was deposited to attach the micromanipulator to the sample. c) The woodpile structure mounted inside the planar microresonator (yellow) used for ferromagnetic resonance measurements with the coordinate system indicating the in‐plane magnetic field angle φ_*H*_. For φ_*H*_ = 0° the external field **H** is parallel to the top‐row of the Ni nanotubes in the woodpile. d) FEM mesh of the woodpile model used in the comsol micromagnetic simulations. The structure consists of eight sublayers of periodically stacked magnetic nanotubes, arranged with a horizontal spacing of *a*_*xy*_ = 1 μm and a vertical spacing of $a_{z}=\sqrt{2}a_{xy}\approx 1.41$ μm. Each has a length of *L* = 4.31 μm and an elliptical cross‐section with semi‐axes *h* = 350 nm and *r* = 125 nm, respectively. The ends are capped with rounded terminations of depth *r*, as shown in the inset. The thickness *d* is assumed to be uniform throughout the structure and is set to *d* = 30 nm.

The outer dimensions of the woodpile shown in **Figure** **1** are 11.7 × 11.7 × 8.4 $\mu m^{3}$, and the in‐plane lattice constant is *a*_*xy*_ = 1 μm. The elliptical cross‐section of each Ni nanotube measures 700 nm × 250 nm. The nanotubes are terminated by ellipsoidal ferromagnetic end‐caps, characterized by a semi‐major axis *h* = 350 nm and a semi‐minor axis *r* = 125 nm, as illustrated in the geometric model in Figure 1d.

Figure 1a–c illustrate the integration of the 8.4‐μm‐high woodpile nanostructure into a planar microresonator fabricated on a separate substrate. The process begins with Xe‐plasma focused ion beam (FIB) etching (Figure 1a), which detaches the woodpile from its original silicon support. The structure is then attached to a micromanipulator needle (Figure 1b) and fully released for transfer. The purple regions denote the deposited Pt used as a mechanical stabilizer (‘adhesive'), while blue indicates the conformal ALD‐grown Ni coating on both the 3D architecture and the silicon substrate. Figure 1c shows the woodpile positioned within the microresonator coil, along with the coordinate system used to define the in‐plane magnetic field angle φ_*H*_ of **H** with respect to the long axis of the top row of nanotubes.

In order to gain microscopic insight into the woodpile's magnetostatics and magnetization dynamics, and to subsequently identify the various resonant modes, we simulated the 3D nanostructure using the finite‐element method (FEM) software package comsol multiphysics.^[^ ^34^ ^]^ This approach allows us to capture spatially varying magnetic fields throughout the complex geometry of the woodpile structure.

Full 3D micromagnetic simulations impose significant demands on the computational resources of the state‐of‐the‐art infrastructure used in this work. For the woodpile model, we therefore simulated only eight vertically stacked layers of nanotubes (i.e., two woodpile‐type unit cells) and seven layers along the horizontal direction. The tetrahedral FEM mesh for the simulations is depicted in Figure 1d. The mesh size varies depending on the local curvature and in the vicinity of the junctions. Despite the reduced total number of nanotubes compared to the real sample, the vertical stacking symmetry and the overall lattice geometry are preserved to ensure consistency with the experimentally realized structure.

**Figure 2:**
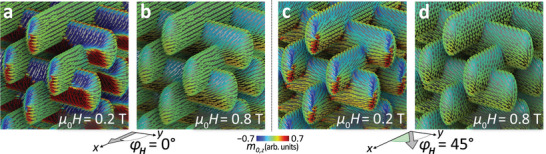
Simulations of the static magnetization configurations for selected in‐plane field strengths and orientations: a,b) along the *x*‐axis (φ_*H*_ = 0°) and c,d) rotated by 45°. The cones represent the local equilibrium direction of the magnetization, **m**_0_, after relaxation, while the color scale indicates its normalized out‐of‐plane component, *m*_0,*z*_.

**Figure** **2** shows the simulated static magnetization configurations in the Ni shell at magnetic fields of 0.2 and 0.8 T applied at angles φ_*H*_ of zero and 45°, respectively. These field values are below and above the maximum shape anisotropy field in Ni, which amounts to μ_0_ *M*_s_ = 0.6 T. Accordingly, noncollinear spin structures in the tubular segments and curved nanocap regions—such as onion states and vortex cores^[^ ^5^, ^20^ ^]^—are prominent for the lower field, transitioning into an almost fully aligned (saturated) configuration at the higher field value of 0.8 T. The fundamental structural dimensions—such as the Ni shell thickness, tubular cross‐section, top‐down symmetry, and lattice periodicity—are consistent with the investigated sample. Our static and dynamic simulations therefore capture the essential magnetostatic and dynamic behavior of the experimentally studied woodpile structure, enabling a direct comparison with the measured FMR spectra.

### 2.2 Extended and Localized 3D Magnon Modes at 14.26 GHz

The ferromagnetic resonance (FMR) measurements were performed using a home‐built spectrometer based on a bridge‐type detection scheme in reflection mode.^[^ ^35^, ^36^ ^]^ A microwave frequency of 14.26 GHz was applied to the resonator antenna to probe the microwave absorption of the 3D Ni structure via inductive coupling. This coupling is significantly enhanced due to the large filling factor within the loop of the planar resonator, thus boosting its detection sensitivity far beyond conventional cavities. At the center of the loop, the RF magnetic field points along the *z*‐axis (normal to the plane) and is oriented perpendicular to the long axes of the Ni nanotubes forming the 3D woodpile.

**Figure 3:**
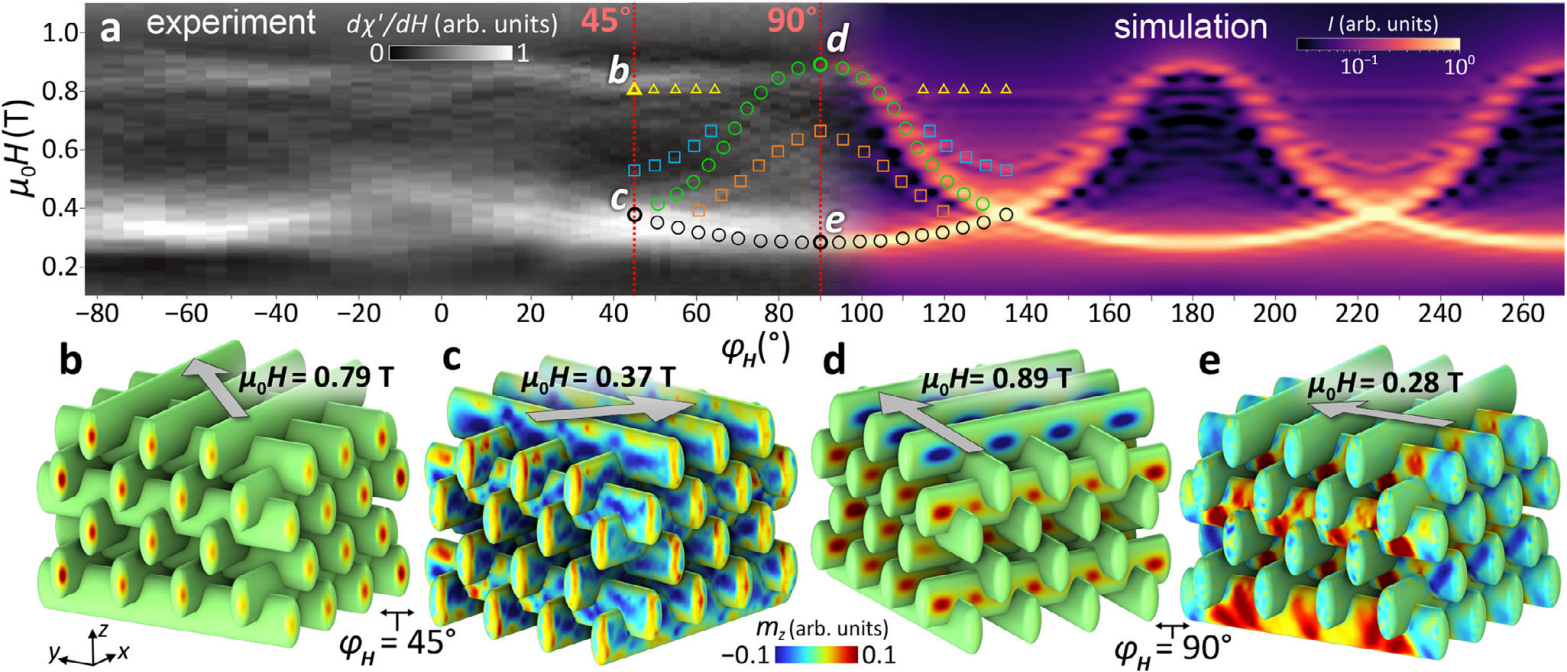
a) The angular dependence of the resonance field at 14.26 GHz reveals a multitude of spin‐wave modes, captured experimentally (grayscale, left‐hand side) and in micromagnetic simulations (color scale, right‐hand side). Resonances are displayed as bright features. Symbols highlight characteristic magnon‐mode branches serving as visual guides. Italic letters denote resonances, whose spatial distributions of the normalized out‐of‐plane component of the dynamic magnetization, *m*_*z*_, are shown in (b–e).

The FMR signals were measured at *f* = 14.26 GHz by sweeping the external in‐plane field **H** at different angles φ_*H*_. The left‐hand side of **Figure** **3**a shows the experimental FMR spectra, displayed as a grayscale intensity map as a function of the in‐plane field angle. For each angle, the external magnetic field was swept from saturation (μ_0_ *H* = 1.2 T) down to zero. Due to the use of lock‐in amplification with field modulation, the measured signal corresponds to the field‐derivative of the FMR response, in contrast to the Lorentzian‐like susceptibility spectra obtained from micromagnetic simulations. To facilitate direct comparison, we plot the real part of the derivative, ℜ(∂χ/∂*H*), which highlights the symmetric, peak‐like features of the resonant absorption. In this representation, white regions indicate strong spin‐precessional absorption in the 3D Ni structure.

The spectra reveal multiple resonance branches with varying signal strengths. Some exhibit a pronounced angular dependence of their resonance field, *H*_res_, with a near‐90° periodicity, while others remain nearly angle‐independent. A cluster of strong resonances appears around 0.3 T for most angles between −80° and 80°. Another prominent group emerges at higher fields, between 0.8 and 0.9 T. These regions are interconnected by several weaker branches that intersect near 0.6 T at φ_*H*_ ≈ 0° and 90°, exhibiting both positive and negative slopes *dH*_res_/*d*φ_*H*_. Notably, the coherent driving of the 3D magnonic crystal by a microresonator results in a much richer and sharper spectral structure than in preliminary experiments, where incoherent magnons at the top surface were explored by inelastic light scattering over a broad range of wave vectors *k*.^[^ ^32^ ^]^

Before discussing selected resonance branches and their associated spin‐precessional dynamics, it is instructive to first perform a quantitative comparison between the experimentally measured and simulated resonance fields. The right‐hand side of Figure 3a contains the simulated data, overlaid with symbols marking selected mode branches extracted from the micromagnetic simulations. The simulated branches cover the same field range as the measured ones, i.e., from about 0.3 to 0.9 T. Upon closer examination—and in agreement with our experimental observations—the simulated spin‐wave modes can be broadly classified into two categories: i) flat branches, which are largely angle‐independent (indicated by triangles), and ii) curved branches, which exhibit pronounced angular variation (marked by circles and squares). The good quantitative agreement between the simulated and measured resonance fields, along with the consistent angular trends, provides strong support for the subsequent analysis of the corresponding spin‐precessional mode profiles and their assignment to the observed spectral features.

The flat branches include multiple resonances at different magnetic field values and are observed near the yellow triangles in both the experimental and simulated spectra, particularly around φ_*H*_ = 40° and 130°, respectively. These modes exhibit minimal sensitivity to changes in the field angle, suggesting a localized character. The micromagnetic simulations confirm that this high‐field mode, labeled *b*, is indeed confined to the cap regions of the structure (see the mode map in Figure 3b). At φ_*H*_ = 45°, the simulations further reveal a whole family of cap‐localized spin‐wave modes, which will be discussed in more detail below.

For fields below 0.47 T, the precessional motion extends deeper into the bulk of the woodpile structure. As shown in Figure 3c, the highest dynamic intensities are observed in the flat sections of the nanotubes, and the mode is no longer confined under a small applied field at φ_*H*_ = 45°.

The pronounced resonances observed in the simulations at φ_*H*_ = 90° and 180° in Figure 3a occur when the external magnetic field is aligned along the long axis of the nanotubes—that is, along either the *x*‐ or *y*‐axis in the simulation coordinate system. Depending on whether the resonance field is high or low, the corresponding modes are spatially excited in different regions, as illustrated in Figure 3d,e, respectively. For lower μ_0_ *H*_res_ (Figure 3e), the modes reside predominantly along the bulk of the nanotubes that are aligned with the applied field. The reduced resonance field in this regime is consistent with a magnetization aligned along an easy axis, as described in Ref. [37]. Supporting this interpretation, a comparable mode and *H*_res_ were also identified in simulations of a single Ni nanotube subjected to a longitudinal magnetic field **H** (see Figure S4, Supporting Information for details). Naturally, in contrast to the isolated nanotube, the modes in the 3D woodpile structure are more complex due to the intersections of orthogonal nanotube segments and the dipolar coupling between neighboring tubes in the lattice. Consequently, the uniform excitation results in characteristic checkerboard‐like interference patterns, induced by the scattering of the coherently excited spin waves at intersections and tube ends. At a large field of 0.89 T (Figure 3d), the resonant spin precession happens mainly in the nanotube segments, which are perpendicular to the applied field, i.e., **H** points into the hard‐axis direction. The excitation is concentrated in the region experiencing the strongest demagnetizing field.^[^ ^7^ ^]^ This occurs because, at higher fields, the static magnetization aligns nearly parallel to the applied field (see Figure 2b,d), and the demagnetizing field peaks in regions where the local surface normal is parallel to the magnetization vector **M**. Modes *d* and *e* (Figure 3) are in fact bulk excitations. At φ_*H*_ = 45°, they become degenerate and merge into mode **c**, as expected from symmetry considerations. Consequently, all modes observed at φ_*H*_ = 45° and at fields above the degeneracy point (0.37 T) must originate from the edge regions, specifically the nanocaps. This distinction is explored in more detail in the following section.

**Figure 4:**
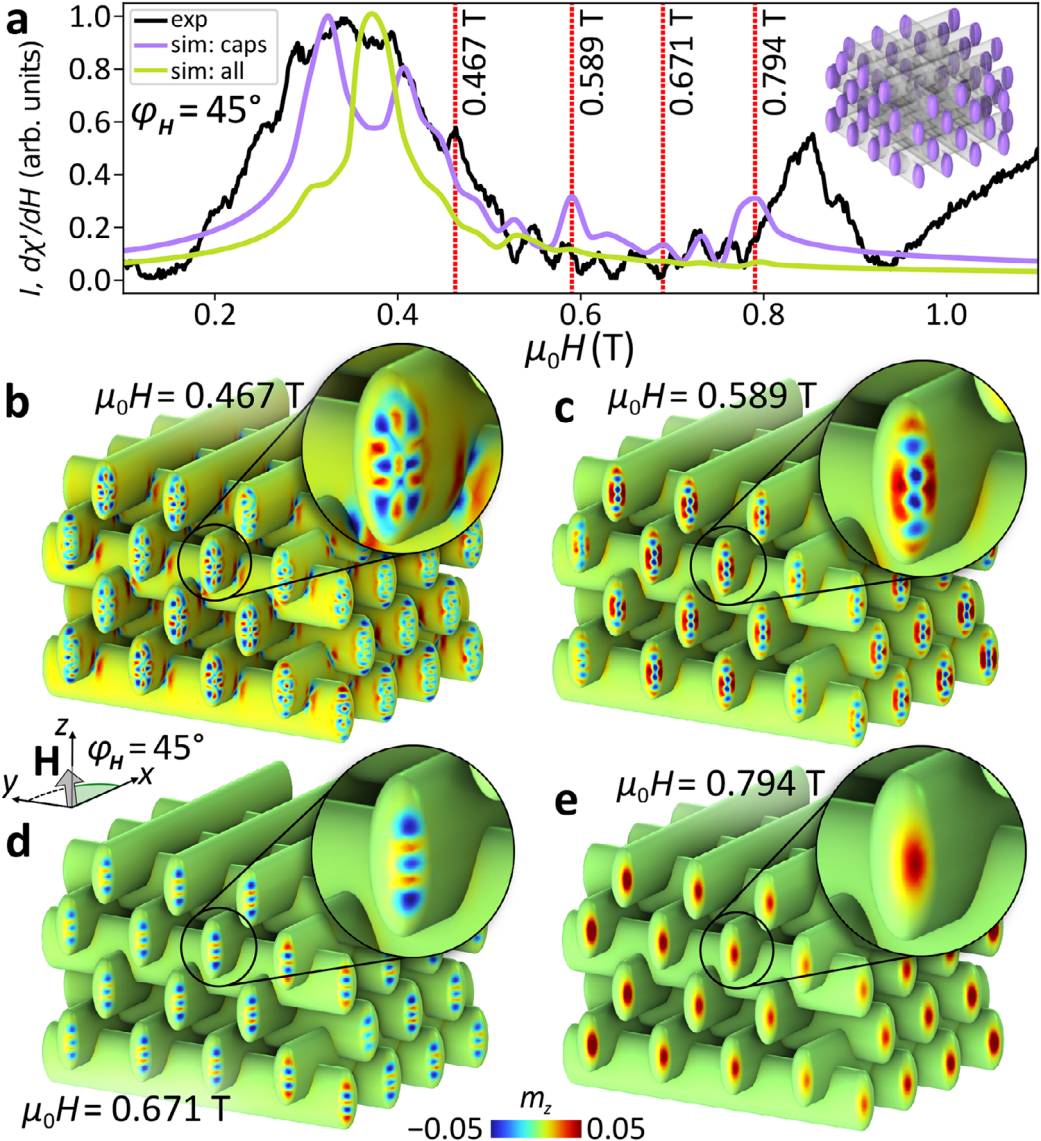
Localized modes at the tube caps. a) FMR spectra at φ_*H*_ = 45° and excitation frequency *f* = 14.26 GHz, obtained from both simulation and experiment. The green curve corresponds to simulated intensities integrated over the entire woodpile structure, see Equation (1), while the purple curve reflects integration restricted to the cap regions (highlighted in the inset, ≈255 nm inward from each edge). The experimental spectrum recorded at the same angle is shown in black. Each curve in panel (a) is independently normalized to its own maximum intensity; therefore, no direct comparison of absolute amplitudes between simulation and experiment is implied. b–e) Simulated spin‐wave mode profiles in the woodpile structure for selected field values. Each panel displays the spatial distribution of the normalized out‐of‐plane dynamic magnetization component, *m*_*z*_, highlighting the localization and geometry of cap modes.

Notably, the conformally coated woodpile structure supports a distinct class of high‐field spin‐wave modes that are spatially confined to the curved end‐caps of the nanotubes as depicted in Figure 3b for φ_*H*_ = 45°. We identify four modes with relatively high intensity between 0.47 and 0.79 T. Their mode maps are shown in **Figure** **4**b–e. All these resonant modes are localized in regions where the demagnetizing field is strongest. As μ_0_ *H* increases beyond 0.47 T, the modes become increasingly confined (Figure 4c–e). With increasing external field, these modes evolve toward the modes with higher‐order spatial profiles to maintain the same resonance frequency of 14.26 GHz – indicating a reduction in mode energy with increasing field. At 0.79 T (Figure 4e), the confined spin‐wave profile exhibits a nearly uniform, spot‐like localization at each cap (same as in Figure 3b). In contrast to that, at 0.47 T (Figure 4b), the simulated spin‐precessional profile reveals a more complex mode with a nontrivial spatial interference pattern and extended dynamic localization at the caps.

Owing to the spatial confinement to a limited volume, their contribution to the simulated FMR spectrum is diminished when averaged over all cells. Consequently, the corresponding spectrum (green curve in Figure 4a) exhibits a markedly narrower linewidth for the peak between 0.35 and 0.45 T compared to the experimental spectrum (black curve), which instead appears as a superposition of multiple overlapping peaks spanning 0.25 to 0.5 T.

To account for these side peaks, we computed a separate spectrum from the micromagnetic simulations by isolating the dynamic contributions of only the tube ends (purple curve in Figure 4a). The integration volume in Equation (1) was restricted to the nanotube end‐cap regions on each side of the structure, as indicated by the purple clouds in the inset of Figure 4a. This targeted approach selectively captures the resonances arising from the caps, revealing two distinct modes in the low‐field regime at 0.32 and 0.41 T. A corresponding cap‐only analysis was also conducted for φ_*H*_ = 90°, which is shown in Figure S1 (Supporting Information).

In the experimental spectrum (black curve in Figure 4a), there is a further pronounced resonance peak visible in the high‐field regime between 0.8 and 0.9 T. This feature is absent in the spectrum accumulated over the full woodpile structure (green curve). Interestingly, while this mode is barely visible in the full‐structure simulation, it appears as the pronounced peak at 0.79 T in the cap‐only spectrum, closely matching the experimental signal. The modes in Figure 4b–d appearing below 0.79 T correspond to the higher‐order cap excitations, producing multiple local maxima in the purple curve. Overall, the experimental spectrum aligns more closely with the cap‐only simulation than with the full‐volume one.

The detection of cap modes, despite being relatively weak in the simulation, highlights the excellent sensitivity of our microresonator‐based FMR detection scheme. We attribute this to the spatial distribution of the microwave magnetic field within the coil (see Figure S3, Supporting Information), which plays a crucial role in mode excitation and detection. While the simulations assume uniform excitation across the entire 3D structure, the experimentally generated magnetic field from the micro‐loop is approximately 2.5 times stronger at the edges and corners than in the central region. This non‐uniform field distribution provides a compelling explanation for the enhanced visibility of cap modes in experiment. There are additional resonance peaks visible in the experimental spectra of Figure 3a between 0.5 and 0.8 T, which can be also observed in the black curve in Figure 4a. These features are distinct from the much lower noise level that can be identified above 1.0 T and the linear increase above 0.95 T, which is an artifact of our setup. Some of the experimental features might result from sample inhomogeneity. We note, however, that the simulated spectrum for the ensemble of cap regions in Figure 4a (purple curve) contains several weak additional resonances as well.

It is important to note that we do not directly compare absolute mode intensities between simulation and experiment. The fundamentally different excitation mechanisms and selection rules—uniform field excitation in simulations versus spatially inhomogeneous microwave fields in the experiment—preclude a quantitative correspondence in mode amplitudes. Rather, the experimental spectrum can be viewed as a weighted superposition of responses from different regions of the structure, effectively combining features captured in both the full‐structure and the cap‐only spectrum. This interplay is particularly evident in the spectra shown in Figure 4a, where distinct contributions from both the extended (bulk) and cap‐localized modes collectively shape the measured signal (black curve).

### 2.3 Phase‐Coherent Spin Precession in Curved Nanocaps

**Figure 5:**
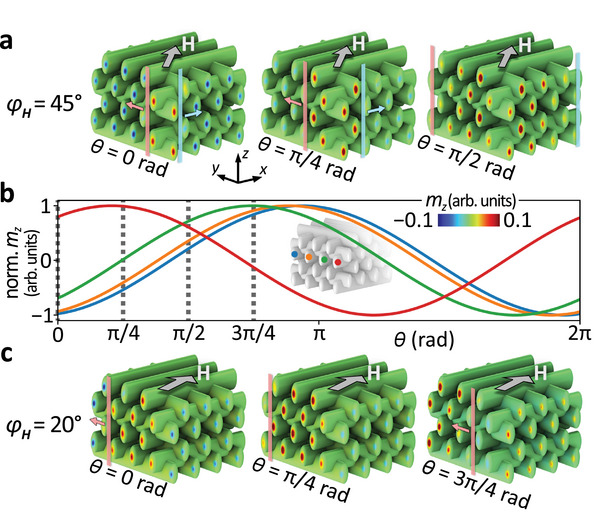
Phase‐dependent spin‐wave dynamics of cap‐localized modes. The modes exhibit wave‐like propagation across the side facets of the 3D woodpile structure, visualized here for two selected in‐plane field orientations and dynamic phases at *f* = 14.26 GHz. a), Time‐resolved snapshots of the out‐of‐plane dynamic magnetization component *m*_*z*_ at three distinct phases, θ = 0, π/4, and π/2 rad of the exciting microwave field, for an applied magnetic field of 0.77 T oriented at φ_*H*_ = 45°. The red and blue vertical bars depict the propagation of the phase fronts. The observed periodic pattern reflects coherent propagation of the cap mode across the structure. b), Phase‐dependent visualization of the normalized magnetization component *m*_*z*_ in selected caps (highlighted in the inset), with colors linking each reference cap to its corresponding curve. c), Same representation as in (a), but for a reduced field angle of φ_*H*_ = 20° and phases θ = 0, π/4, and 3π/4 rad. Together, the panels demonstrate the dynamic character and spatial coherence of the cap‐localized spin‐wave modes.

To probe the coherence of the cap modes, we examine whether they precess in phase, as one might expect under the uniformly applied microwave field assumed in the simulations. **Figure** **5**a shows the spin‐precessional amplitudes extracted from the dynamic simulations at three points in time for *f* = 14.26 GHz and φ_*H*_ = 45°. Surprisingly, the different caps reach their maximum spin‐precessional amplitudes at different times (Figure 5b), indicating that the excitation is not uniformly phased across the structure. The four caps analyzed in Figure 5b are positioned along a central row, as shown in the inset, with colors corresponding to the respective amplitude traces. It is important to emphasize that these plots show normalized magnetization amplitudes—each trace is individually scaled to its own maximum. As a result, the curves convey only the relative phase evolution, without reflecting differences in absolute amplitude between the caps. Nevertheless, they clearly reveal that the dynamic magnetization in the caps exhibits a wave‐like phase gradient across the structure along its lateral edges. Vertical lines in Figure 5a serve as visual guides, illustrating the temporal evolution of the phase across two adjacent side facets. Animations of this behavior are included in the Supporting Information.

This counter‐intuitive behavior is also observed at φ_*H*_ = 20° (Figure 5c) for the same magnetic field value of 0.77 T. Similar to the case of φ_*H*_ = 45°, the spins in the caps exhibit a continuous phase shift across the surface of the woodpile structure, and their amplitudes follow the same normalized profiles as shown in Figure 5b. In the φ_*H*_ = 20° field configuration, the wave‐like character of the phase front becomes even more pronounced, particularly on the side of the structure oriented more perpendicular to the applied magnetic field **H**.

To better understand the physical origin of this effect, we performed additional simulations on a simplified chain of identical Ni nanotubes terminated by curved caps, described in Section S6 (Supporting Information). These toy‐model calculations allowed us to isolate the essential dipolar coupling mechanism responsible for the observed phase evolution. The distributions of the dynamic magnetic scalar potential around the caps reveal that when the field is applied parallel to the nanotube axis, the dynamic potential alternates in sign between neighboring caps (+− + − + −), forming a configuration that minimizes the instantaneous dipolar‐field energy (see Figure S5, Supporting Information). Rotating the field away from this direction distorts this pattern, leading to regions of equal sign of the magnetostatic potential that locally increase the dipolar energy. The system compensates by dynamically reorienting the relative precessional phases of adjacent caps, which manifests as the observed phase flow across the structure.

Furthermore, the strength of this phase correlation strongly depends on the inter‐cap spacing. As demonstrated in Figure S6 (Supporting Information), increasing the distance between neighboring nanotubes systematically reduces the phase shift amplitude until the effect eventually vanishes for sufficiently large separations. This trend confirms that the phenomenon arises from dynamic dipolar coupling rather than from local variations in curvature or demagnetizing fields. In the actual woodpile geometry, the characteristic spacing between neighboring caps ensures a coupling strength sufficient to sustain phase coherence, leading to the wave‐like evolution observed in Figure 5.

Interestingly, the corner cap—lacking neighbors on one side—shows a distinct phase offset compared to those in the bulk of the array (see red trace in Figure 5b). This behavior reflects the asymmetric local dipolar‐field environment at the structure's corner (see Figure S5, Supporting Information) and provides a natural analogue of local geometric perturbation.

Our findings underscore a key result: the curved nanocaps support collective, dipolarly coupled, cap‐localized spin‐wave modes with coherent, field‐dependent phase relationships. The spatial coherence across neighboring caps is clearly resolved, revealing a propagating phase gradient that emerges despite the uniform microwave excitation. This emergent phase ordering arises from the interplay between the geometry‐induced localization of spin‐wave modes, their long‐range dynamic dipolar coupling and the finiteness of the structure that introduces disturbance into the system dynamics.

This observation might reflect an alternative realization of the unusual nature of confined modes in chiral magnetic systems.^[^ ^38^ ^]^ More broadly, this observation suggests that even within complex 3D nanostructures, the mutual dipolar coupling between neighboring elements can give rise to well‐defined phase relationships. While the degree of controllability remains to be demonstrated experimentally, the existence of such coherent phase correlations under uniform excitation indicates that 3D magnonic systems may naturally support mechanisms for phase‐based information encoding and guided signal transfer. In this sense, the observed effect can be regarded as an initial step toward exploring phase coherence as a functional degree of freedom in volumetric magnonic architectures.

### 2.4 Resonance Fields and Their Magnon Modes Beyond Shape Anisotropy Fields

**Figure 6:**
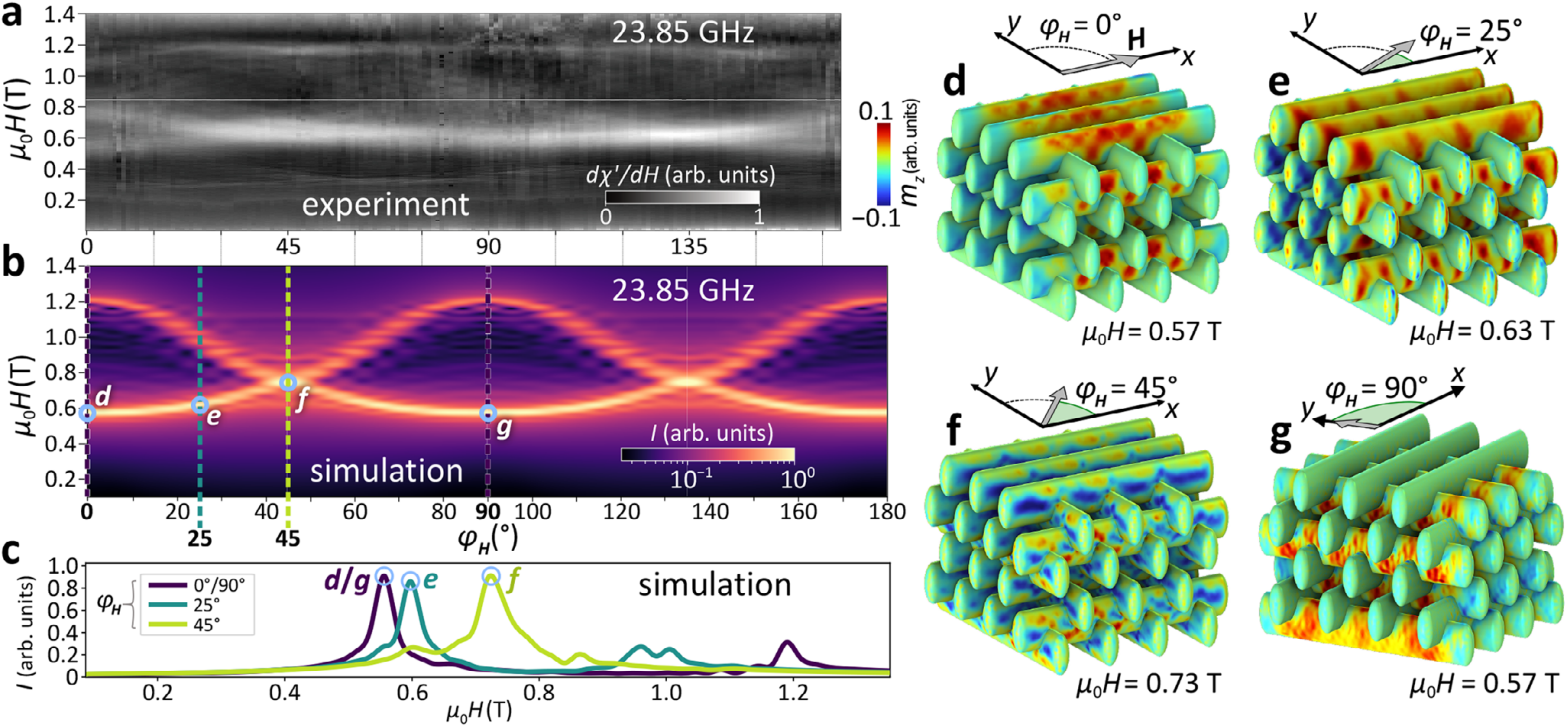
FMR experiment and simulations at 23.85 GHz. a) Experimentally measured FMR spectra as a function of magnetic field strength, recorded at various in‐plane field orientations φ_*H*_ for an excitation frequency of 23.85 GHz. The field was swept from 1.4 T down to zero, with white color corresponding to maximum absorption. b) Simulated angular‐resolved spectra reproduce the key features observed in the experiment. Dashed lines and letters mark selected field angles, for which panel (c) shows the corresponding simulated FMR spectra, enabling clearer identification of mode branches. d–g) The spatial profiles of the normalized out‐of‐plane dynamic magnetization component *m*_*z*_ for the labeled resonances illustrate the diversity of spin‐wave character in the 3D woodpile structure. For φ_*H*_ = 0°, the applied field is aligned parallel to the top row of the Ni nanotubes.

The minimum resonance field observed in the experiments presented so far is approximately 0.3 T, which lies below the maximum shape anisotropy field of the ALD‐grown polycrystalline Ni, estimated to be 0.6 T. To understand the influence of shape anisotropy fields, we performed additional measurements at a much higher frequency of 23.85 GHz. Although our microresonator was optimized for a microwave resonance at 14.26 GHz, we were still able to operate it off‐resonantly. For these measurements, a Mach–Zehnder‐type microwave interferometer was added to the signal path of the FMR spectrometer. This reduced the very high idle off‐resonant reflection of the microresonator by more than 35 dB; thus, we avoided saturation of the low‐noise amplifier. The grayscale graph in **Figure** **6**a combines the spectra taken from high to small magnetic fields at different angles φ_*H*_.

As expected, the resonances observed at 23.85 GHz appear at higher magnetic fields μ_0_ *H*_res_—above 0.5 T due to the increased excitation frequency. The most prominent features are centered around 0.6  and 1.2 T, with several weaker branches connecting them across the intermediate field range. Ni nanowires studied at almost the same frequency in Ref. [39] showed only two branches for which *H*_res_ followed identical 180‐degree rotational symmetries.

While the resonance branches that we observe at 14.26 and 23.85 GHz share overall similarities—including a consistent shift of resonance fields with increasing frequency—a difference arises in weaker spin‐precessional amplitudes consistent with the higher‐frequency excitation, where the magnetic susceptibility is reduced and the number of prominently resolved modes at φ_*H*_ = 0° in the experimental data increased, as seen by comparing Figure 3a and Figure 6a. Four distinct branches are clearly visible near 0.55, 0.75, 1.1, and 1.2 T. We attribute this improved mode resolution and the narrow linewidths to the larger applied field, which facilitates saturated magnetic states across all branches. Interestingly, the authors of the previous study on incoherent magnons^[^ ^32^ ^]^ identified their richest spectral characteristics and most well‐separated modes also when the magnetic field was aligned with the long axis of the top‐layer nanotubes. By spatially resolved spectroscopy, three distinct modes were observed at zero angle in Figure 4 of that work. The three modes were attributed to the center‐, tube‐end‐, and corner‐regions of the top surface of the 3D woodpile structure, respectively.

The simulations shown in Figure 6b represent power spectra integrated over the entire 3D woodpile volume. Three representative line cuts are displayed in Figure 6c. At φ_*H*_ = 0°, the simulations predict fewer prominently resolved branches compared to our experimental results. In the dark violet curve of Figure 6c, only two clear peaks are observed. This discrepancy can be attributed to the spatial averaging inherent to the simulation approach, which dilutes the contribution of modes that are strongly localized in the cap regions. Along the low‐field branches, the spatial mode profiles (Figure 6d–g) closely resemble those found at 14.26 GHz (Figure 3b–e), indicating that the fundamental nature of the excitations remains largely unchanged. As before, we observe a coexistence of bulk‐like and cap modes, governed by field orientation and geometric confinement. Despite the reduced amplitude, the cap dynamics at 23.85 GHz reveal a more pronounced wave‐like behavior. This is attributed to the shorter effective wavelength (i.e., larger wave vector) at higher frequencies, which leads to faster spatial oscillations and more pronounced phase fronts across the caps (see Section S6, Supporting Information). As a consequence, the phase coherence between neighboring caps becomes more apparent, reinforcing the interpretation of these modes as coherent, propagating edge excitations.

Taken together, these findings support the interpretation that microresonators exhibit excellent sensitivity to cap‐localized magnon modes—modes that are of particular interest in the context of 3D topological magnonics.

## 3 Conclusion

In summary, we have successfully integrated a full‐fledged 3D magnonic crystal into a microresonator, enabling the coherent excitation and detection of its magnon modes. The angular dependence of the spectra at the resonator frequency of 14.26 GHz revealed a clear fourfold symmetry (90‐degree rotational symmetry), consistent with the in‐plane geometry of the woodpile lattice. We experimentally observed a rich set of spin‐wave modes, reflecting the complex unit cell of the fcc 3D structure. Our micromagnetic simulations based on the same geometry showed a good quantitative agreement with the measured resonance fields at both 14.26 and 23.85 GHz.

By visualizing the simulated mode profiles, we identified both extended modes spanning the full 3D network and localized modes confined to the curved nanocaps. These cap modes, resolved in both simulation and experiment, showed up in flat branches. They exhibited robustness against variations in the magnetic field orientation and display a directed phase evolution under uniform excitation—intriguing features that may suggest topological characteristics.

Interestingly, the microresonator loop exhibited enhanced sensitivity to such edge magnon modes. This work establishes a versatile platform for studying coherent spin dynamics in 3D magnonic systems and highlights the potential for further engineering of their band structure. It also demonstrates a practical approach to integrating complex 3D magnetic architectures into functional microwave devices.

## 4 Experimental Section

### Sample Fabrication

The 3D polymer nanoscaffold for the woodpile was fabricated by using a two‐photon lithography system (Nanoscribe Photonic Professional GT+) at the Center of MicroNanoTechnology at EPFL. A droplet of IP‐Dip2 photoresist (refractive index *n* = 1.547) was deposited onto a silicon substrate (10 × 10 mm^2^, 700 μm thick). The dip‐in laser lithography configuration was used with a 63× magnifying objective and infrared femtosecond laser (780 nm) for localized polymerization in PiezoScan mode. The laser power was adjusted to 10 mW during writing. Following the exposure, the substrate underwent a sequential development process by initial immersion in propylene glycol monomethyl ether acetate for 20 min to remove unexposed resist. Then the structures were rinsed for 5 min in isopropyl alcohol and dried in a nitrogen flow. Finally, the substrate, along with additional bare silicon reference substrates, was placed inside the ALD chamber of a Beneq TFS200 system at CMi. A 5‐nm‐thick Al_2_O_3_ layer was first deposited, followed by a 30‐nm‐thick ferromagnetic Ni layer using nickelocene as a precursor. The details of the ALD deposition process are described in Refs. [32, 33]. The choice of silicon as substrate was intentional to minimize charging effects during the subsequent FIB etching processes.

To transfer the woodpile into the microresonator, Xe‐plasma FIB etching was used by means of a FEI Helios G4 plasma‐FIB UXe system at the Interdisciplinary Center for Electron Microscopy at EPFL. It combines a FIB with a SEM. To facilitate the detachment of the woodpile structure from the Si substrate, three trenches were defined by FIB milling on three adjacent sides of the structure. A micromanipulator needle was then attached to the top corner of the woodpile by depositing a Pt patch (see Figure 1a). To remove the Si substrate from the base of the 3D structure, the fourth side was subsequently cut at the bottom of the woodpile. A 30 kV ion beam was employed with currents ranging from 15  to 1 nA, depending on the proximity to the structure's base. After positioning the nanostructure inside the omega‐shaped thin‐film microresonator coil, it was secured by spot‐deposition of Pt at two bottom corners and cut off the manipulator needle by Xe FIB.

### Microresonator Ferromagnetic Resonance

The planar microresonator design was optimized for an eigenfrequency of about 14.26 GHz and 50 Ω impedance using the ansys hfss simulation package.^[^ ^40^, ^41^ ^]^ The nominal inner loop diameter was 20 μm. It was prepared on a highly resistive Si(001) substrate by means of photolithography, molecular‐beam epitaxy of Cr/Cu/Au, and subsequent lift‐off processing.^[^ ^35^, ^42^, ^43^ ^]^ The metallic loop antenna had a total thickness of about 700 nm. In the center of the loop the RF magnetic field was along the *z*‐axis (out of plane) and perpendicular to the long axes of the Ni nanotubes forming the 3D woodpile. Simulations of the RF‐field distribution are given in Figure S3 (Supporting Information).

The FMR measurements were performed using a home‐built spectrometer in reflection mode.^[^ ^35^, ^36^ ^]^ The signals were taken as a function of the in‐plane field **H** applied at different angles φ_*H*_. At every angle, the average of 25 field sweeps was taken to reduce the noise. An additional field‐modulation with 1 mT amplitude at 78 kHz was employed to allow for lock‐in detection. The residual noise level is visible in Figure 4a above 1.0 T. The detection limit of this 20‐μm‐diameter resonator was estimated to be around 10^8^ Ni spins.

The measurements at 23.85 GHz were far off the eigenfrequency of the resonator. This results in the idle resonator reflecting approximately 20 dB more power, which would lead to saturation of the low‐noise amplifier (LNA) circuit. To mitigate this issue, an interferometric approach that enabled cancellation of the idle signal prior was employed to entering the LNA. This was achieved by splitting the microwave signal and introducing a π phase shift along with careful attenuation to one of the paths. The two signals were then recombined using the Mach–Zehnder interferometer principle, effectively canceling more than 35 dB of the background trace and allowing mainly the resonance signal to be passed to the LNA and detector. This avoids saturating the low‐noise pre‐amplifier. Thereby, measurements with a large signal‐to‐noise ratio far off the resonator's eigenfrequency become possible. The microwave power at the sample was set to −10 dBm = 100 μW without the interferometer, and to +4 dBm with the interferometer. Using an IQ‐mixer (in‐phase and quadrature mixer) as detector in conjunction with two lock‐in amplifier channels, the field derivatives of the FMR absorption ∂χ″/∂*H* and dispersion ∂χ′/∂*H* signals were retrieved simultaneously.. In the graphs, the dispersion derivative signal (*d*χ′/*dH*) was shown. A resonance then had a symmetric line shape and appears as a local maximum in an experimental line spectrum and white (bright) in the gray‐scale plots, respectively.

Note that FMR was known to detect the uniform spin precession, i.e., spin waves with *k* = 0, and standing spin‐wave modes. Their wavelength was given, due to confinement, by the dimensions of the sample or due to localization by extrema of the effective field (relevant for edge or cap modes). Only those modes lead to signals, whose dynamic mode profiles spatially had a non‐vanishing projection of the dynamic magnetization parallel to the microwave magnetic field direction. In case of a magnonic crystal, FMR could detect miniband modes, which existed at the Γ point. Such considerations explain the richness of the FMR spectra.

### Micromagnetic Simulations

The FEM‐based comsol multiphysics software package^[^ ^34^ ^]^ was used for the micromagnetic simulations. The *Coefficient Form* interface was employed, a flexible framework that enables the definition and solution of custom partial differential equations. In this study, it was used to implement the Landau–Lifshitz–Gilbert equation [Equation (S1), Supporting Information]. The following material parameters were used for Ni matching our experimental conditions (see Figure S2, Supporting Information): saturation magnetization *M*_s_ = 400 kAm^−1^ as reported for the ALD‐grown Ni,^[^ ^33^ ^]^ exchange stiffness *A*_ex_ = 8 pJm^−1^, and the gyromagnetic ratio γ = 193.6 GHzT^−1^. During each time‐domain (relaxation) simulation, the Gilbert damping constant α was set to a high value to ensure rapid convergence to the equilibrium state. For the subsequent frequency‐domain simulations, it was reduced to α = 0.008. Further methodological details are provided in the Supporting Information and in Refs. [13, 44, 45].

To emulate the FMR condition, a spatially uniform dynamic magnetic field of magnitude μ_0_ *h*_RF_ = 1 mT was applied, polarized along the *z*‐axis (always perpendicular to **H**). By sweeping the static field magnitude and keeping the microwave frequency *f* fixed, the magnetization response throughout the volume was computed. Then, to highlight the global resonance signature, the complex, normalized dynamic magnetization vector, **m** = (*m*_*x*_, *m*_*y*_, *m*_*z*_) = **M**/*M*_s_ was integrated. In particular, on its *m*_*z*_ component was focused, which was oriented perpendicular to the static magnetic field. The resonance intensity is defined as

(1) $$I={\left(\int_{V}ℜ\left\{m_{z}\right\}\;dV\right)}^{2}+{\left(\int_{V}ℑ\left\{m_{z}\right\}\;dV\right)}^{2}$$

This integral captured the overall resonance intensity of the sample by combining the real and imaginary parts of *m*_*z*_ integrated over the entire sample volume *V*. In selected cases, the integration was restricted to the cap regions, defined as the side‐edge sections of the woodpile structure extending approximately 255 nm inward from each edge. An unstructured tetrahedral mesh was generated throughout the ferromagnet and its surrounding region, with finer elements concentrated within and around the Ni shell to accurately resolve the magnetic interactions. The complete 3D model of the woodpile structure (excluding the surrounding region) as shown in Figure 1d consists of approximately 150 000 elements.

## Conflict of Interest

The authors declare no conflict of interest.

## Author Contributions

H.G., K.L., and M.G. contributed equally to this work and wrote the paper with inputs from all authors. H.G., K.L., and D.G. conceived the work. H.G. prepared the samples. K.L. performed and analyzed the FMR measurements. M.G. and M.K. performed and interpreted the numerical simulations. R.N. designed, simulated, and prepared the microresonators. D.G., M.K., and J.L. supervised the project. All authors read and commented on the paper.

## Supporting information



Supporting Information

Supporting Information

## Data Availability

The data that support the findings of this study are available from the corresponding author upon reasonable request.
