# Supplementary material for: Coherent Spin Waves in Curved Ferromagnetic Nanocaps of a 3D‐Printed Magnonic Crystal
Source: Small. 2025 Dec 17;22(7):e08983. doi: 10.1002/smll.202508983 (PMC12862454; doi:10.1002/smll.202508983)
Supplement: Supplementary file 1 — Supporting Information [file SMLL-22-e08983-s002.pdf]

Supporting Information

for *Small*, DOI 10.1002/smll.202508983

Coherent Spin Waves in Curved Ferromagnetic Nanocaps of a 3D-Printed Magnonic Crystal

Huixin Guo, Kilian Lenz, Mateusz Gołębiewski, Ryszard Narkowicz, Jürgen Lindner, Maciej Krawczyk and Dirk Grundler**

— Supporting Information —

Coherent Spin Waves in Curved Ferromagnetic Nanocaps of a 3D-Printed Magnonic Crystal

Huixin Guo[1], Kilian Lenz[2], Mateusz Gołębiewski[3], Ryszard Narkowicz[2], Jürgen Lindner[2], Maciej Krawczyk[3], Dirk Grundler[1,4]

[1]School of Engineering, Institute of Materials, Laboratory of Nanoscale Magnetic Materials and Magnonics, École Polytechnique Fédérale de Lausanne (EPFL), Lausanne 1015, Switzerland.
[2]Institute of Ion Beam Physics and Materials Research, Helmholtz-Zentrum Dresden–Rossendorf, Bautzner Landstr. 400, 01328 Dresden, Germany.
[3]Institute of Spintronics and Quantum Information, Faculty of Physics and Astronomy, Adam Mickiewicz University, Uniwersytetu Poznańskiego 2, 61-614 Poznań, Poland.
[4]School of Engineering, Institute of Electrical and Micro Engineering, École Polytechnique Fédérale de Lausanne (EPFL), Lausanne 1015, Switzerland.

Contents

S1 Comparison of cap modes at $\varphi_H = 90°$ 2
S2 FMR measurement of the Ni-ALD reference sample. 3
S3 Microresonator field distribution 4
S4 Dynamic simulations of the woodpile 4
S5 Simulation of single tubes 5
S6 Phase-resolved dynamics of cap-localized spin-wave modes 7
S6.1 Dynamic magnetic potential in dependence of the field orientation 8
S6.2 Dependence on inter-cap spacing 9

S1 Comparison of cap modes at $\varphi_H = 90°$

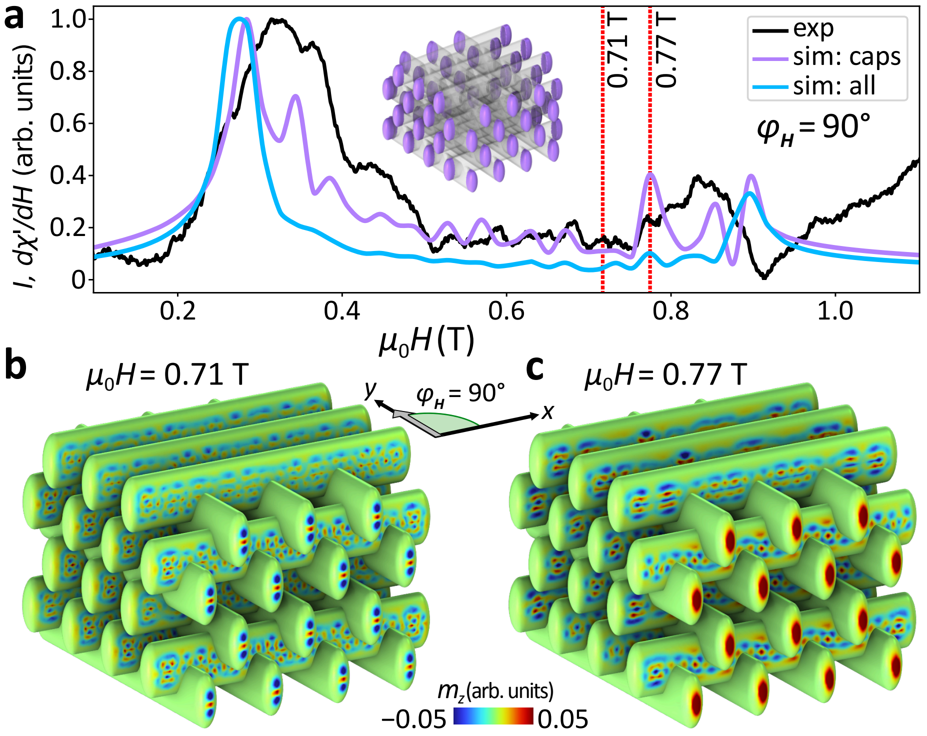

Figure S1 Analogous to the $\varphi_H = 45°$ case shown in Figure 4, this figure presents results for $\varphi_H = 90°$ and excitation frequency $f = 14.26$ GHz, obtained from both simulation and experiment. In (a), the blue curve represents simulated intensities integrated over the entire woodpile structure, see Equation (1), while the purple curve corresponds to integration limited to the cap regions, as indicated in the inset. The experimental spectrum recorded at the same field angle is shown in black. Each curve is independently normalized to its own maximum, and thus no direct comparison of absolute amplitudes is implied. (b) Simulated spin-wave mode profiles for selected magnetic field values, showing the spatial distribution of the normalized out-of-plane dynamic magnetization component, m_z. The images highlight the localization and symmetry of cap modes in the woodpile geometry.

S2 FMR measurement of the Ni-ALD reference sample.

Figure S2 shows the frequency-dependent FMR measurement on the 30-nm-thick Ni-ALD reference film. This sample was processed in the same ALD process. The FMR was measured by vector-network analyzer FMR with the sample placed on a co-planar waveguide. We determined an effective magnetization of $\mu_0 M_{\mathrm{eff}} = 400$ mT, and a g-factor of $g = 2.174(1)$ in strong consistency with previous reports [28,29], confirming the reproducibility and robustness of our approach. The analysis of the linewidth yields a Gilbert damping of $\alpha = 0.025$, which is slightly lower than what was found for thin Ni/Si(001) films [40].

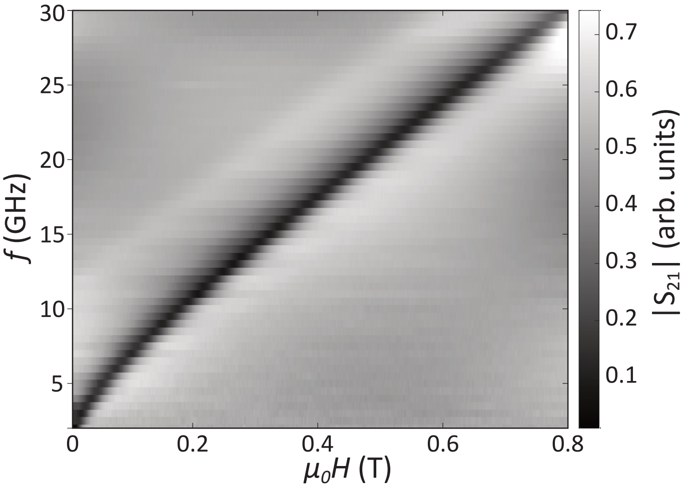

Figure S2 Intensity plot of the magnitude of the scattering parameter S_{21} showing the in-plane frequency-field dependence measured by vector-network analyzer FMR on the Ni-ALD reference thin film.

S3 Microresonator field distribution

Figure S3a shows the field distribution of the rf magnetic field of a bulk Ni cube inside the microresonator. We used Ansys HFSS to simulate this. The dimensions are the same as the woodpile. Figure S3b shows linescans of the field strength at the bottom of the sample and in 10 µm height above the substrate, i.e. at the top of the sample, for two directions, respectively.

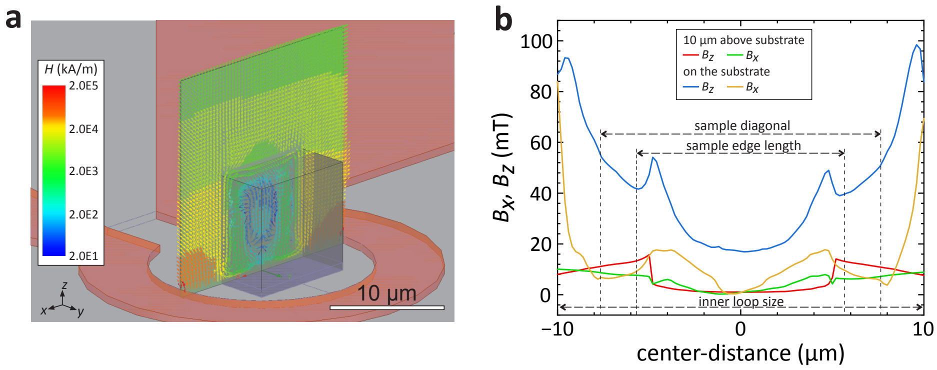

Figure S3 (a) Field distribution within the microresonator loop antenna with respect to the sample (at 14.26 GHz and 0 dBm power) simulated using the Ansys HFSS. (b) Line profiles of the B_x and B_z field strength at the bottom and top of the sample. The arrows mark the sample and antenna dimensions. As the resonator antenna is only 700 nm high, the bottom of the 11.7 µm large sample experiences the strongest RF excitation field of about 20–40 mT compared to the top. The RF field is even stronger at the corner regions of the structure, as they are the closest part to the antenna.

S4 Dynamic simulations of the woodpile

In the micromagnetic framework, we solve the dynamical Landau–Lifshitz–Gilbert equation

$$\frac{\partial \mathbf{M}}{\partial t} = -\gamma\mu_0 \left(\mathbf{M} \times \mathbf{H}_{\text{eff}}\right) + \frac{\alpha}{M_{\text{s}}}\left(\mathbf{M} \times \frac{\partial \mathbf{M}}{\partial t}\right), \tag{S1}$$

where $\mathbf{M}$ is the magnetization vector, μ_0 is the vacuum permeability, and α is the Gilbert damping constant. The effective field $\mathbf{H}_{\text{eff}}$ is given by

$$\mathbf{H}_{\text{eff}} = \mathbf{H} + \mathbf{H}_{\text{d}} + \mathbf{H}_{\text{exch}}. \tag{S2}$$

Here, $\mathbf{H}$ is the externally applied static field, $\mathbf{H}_{\text{d}}$ is the demagnetizing field, and $\mathbf{H}_{\text{exch}} (= \frac{2A_{\text{ex}}}{\mu_0 M_{\text{s}}^2}\nabla^2\mathbf{M})$ is the exchange field. The demagnetizing field $\mathbf{H}_{\text{d}}$ is derived from the magnetostatic potential U_{m}, satisfying

$$\nabla^2 U_{\text{m}} = \nabla \cdot \mathbf{M} \qquad \text{inside the ferromagnet,} \tag{S3}$$

$$\nabla^2 U_{\text{m}} = 0 \qquad \text{outside,} \tag{S4}$$

$$\mathbf{H}_{\text{d}} = -\nabla U_{\text{m}}. \tag{S5}$$

The total magnetostatic potential U_m can be expressed as a sum of its static and dynamic components, $U_\mathrm{m}(\mathbf{r},t) = U_\mathrm{m}^{(0)}(\mathbf{r}) + u_\mathrm{m}(\mathbf{r},t)$, where $U_\mathrm{m}^{(0)}$ denotes the static potential associated with the equilibrium magnetization configuration, and u_m represents the dynamic contribution induced by spin-wave precession. The nonuniform distribution of $\mathbf{M}$ in space and the finite size of the structure make the demagnetizing field particularly relevant for the spin-wave modes.

The simulation workflow involves two stages:

i) *Magnetization relaxation:* A time-domain simulation is performed to relax the magnetization into its static equilibrium under the chosen external field. This step guarantees that we capture the static magnetization profile, including any inhomogeneities arising from geometry and boundary effects. Here, α is set to a high value to ensure rapid convergence to the equilibrium state.

ii) *Dynamic perturbation:* Following relaxation, we carry out a frequency-domain forced-response analysis in which the magnetization is exposed to a small dynamic field uniform in space and oriented along the z-axis. We sweep over a range of static magnetic field magnitudes and in-plane orientations to replicate the experimental protocols. The linear approximation is invoked by decomposing the total magnetization $\mathbf{M}$ into a static equilibrium component and a small dynamic perturbation:

$$\mathbf{M}(\mathbf{r},t) = M_\mathrm{s}\hat{\mathbf{n}}(\mathbf{r}) + \mathbf{m}(\mathbf{r},t), \quad \text{with } \mathbf{m} \perp \hat{\mathbf{n}}. \tag{S6}$$

Here, $\hat{\mathbf{n}}(\mathbf{r})$ is the local unit vector defining the equilibrium orientation of the magnetization, and M_s is the saturation magnetization. The static background configuration for each field setting is inherited from the preceding relaxation step.

To solve for the magnetostatic potential U_m in and around the ferromagnetic region, we embed the entire structure in a sufficiently large computational domain. Dirichlet boundary conditions, $U_\mathrm{m} = 0$, are imposed on the outer boundaries of this domain to mimic the decay of stray fields at large distances. The size of this domain is chosen to be several times larger than the overall dimensions of the woodpile in each spatial direction, ensuring that the numerical solution is unaffected by artificial boundary effects.

S5 Simulation of single tubes

We have performed complementary simulations on individual nanotubes (**Figure S4**), which serve as fundamental building blocks of the woodpile nanostructure analyzed in the main study. Each simulation comprises approximately 100 000 tetrahedral elements.

These simulations were performed to further elucidate the origin of the observed resonance modes. Figure S4a shows the simulated FMR at $f = 14.26$ GHz as a function of the in-plane field angle for a single ferromagnetic Ni tube—the same size used in the woodpile structure. The color scale represents the spin-wave intensity, calculated from the dynamic magnetization component m_z. The resonance field depends strongly on the external field orientation (φ_H) and the shape anisotropy of the tube. Since the tube's long axis constitutes the magnetic easy axis, the resonance field is minimized when $\varphi_H = 0°$. In contrast, when the field is applied perpendicular to

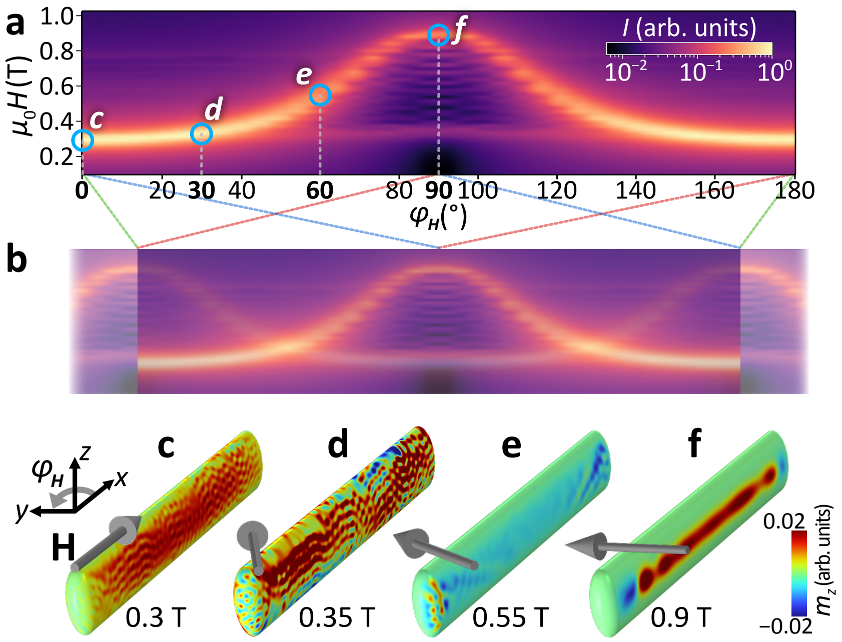

Figure S4 Micromagnetic simulations of a single Ni nanotube under different external field angles φ_H. (a) Simulated FMR angular dependence, showing the resonance intensity in logarithmic scale, as a function of applied field value $\mu_0 H$ and angle φ_H at $f = 14.26$ GHz. (b) Superposition of two single-tube angular dependencies offset by 90°. It illustrates how the woodpile geometry can be approximated by overlapping the resonance spectra of two orthogonal tubes. (c)–(f) Dynamic magnetization distribution maps for $\varphi_H = 0°, 30°, 60°$, and 90°, respectively. Each map was taken from the highest intensity branch of the resonance occurring at different external field values for each angle.

the tube axis ($\varphi_H = 90°$), the magnetization lies along the hard axis, leading to an increased resonance field.

In Figure S4b, we extend the single-tube analysis by overlapping the simulated FMR angular dependencies from two Ni tubes, each rotated by 90° with respect to the other. This superposition effectively corresponds to shifting the original angular dependence by 90° and adding it to the initial plot. This acts as a simplified model of the woodpile geometry, where tubes are arranged in two orthogonal directions. While this visualization provides insight into how the principal resonance features could combine in the full woodpile, it does not account for the actual structural joints between tubes or the additional interactions present in the real sample. Consequently, the complete woodpile spectrum exhibits shifted resonance fields. This shift toward higher fields arises from the increased effective demagnetizing field in the full structure, particularly due to geometric features such as joints and intersections. Nevertheless, the superposition shown in Figure S4b demonstrates that, to a first approximation, the principal resonances of the full woodpile can be interpreted as emerging from the individual nanotube responses.

To present the spatial distribution of the specific modes, Figure S4c–f visualize the dynamic magnetization distribution m_z in the tube for four representative field angles and values. When in Figure S4c $\varphi_H = 0°$, the dynamic magnetization profile is nearly uniform along the tube, consistent with its easy-axis alignment. As φ_H increases, the mode structure becomes more complex, showing enhanced localization near the tube ends. Notably, for the perpendicular orientation ($\varphi_H = 90°$) in Figure S4f, a high-amplitude response in m_z appears at a relatively large external field value of about 0.9 T. This spin-wave mode is strongly localized in the region normal to the field axis. These micromagnetic results confirm that shape anisotropy and demagnetizing fields together govern the FMR response of the Ni tube across different field orientations.

S6 Phase-resolved dynamics of cap-localized spin-wave modes

We provide six `.gif` animations visualizing the phase-resolved spin-wave dynamics of the cap-localized modes in the 3D woodpile nanostructure at excitation frequencies of 14.26 GHz and 23.85 GHz, for in-plane magnetic field orientations of $\varphi_H = 0°$, 20°, and 45°, respectively. The filenames are as follows:

- `CapModes-14GHz-0deg-0_77T.gif`
- `CapModes-14GHz-20deg-0_77T.gif`
- `CapModes-14GHz-45deg-0_77T.gif`
- `CapModes-24GHz-0deg-1_1T.gif`
- `CapModes-24GHz-20deg-1_1T.gif`
- `CapModes-24GHz-45deg-1_1T.gif`

Each animation presents a continuous evolution of the dynamic magnetization phase, offering a time-resolved perspective on the propagation of spin-wave modes confined to the lateral cap regions of the structure. The visualized quantity is the out-of-plane dynamic magnetization component m_z, capturing the spatially resolved precessional behavior across the caps as a function of dynamic phase.

At 14.26 GHz, the cap modes exhibit clear wave-like propagation across the side facets, characterized by coherent phase fronts and well-defined oscillatory patterns. At the higher excitation frequency of 23.85 GHz, these modes remain localized to the caps, but the confinement becomes more pronounced, occupying an even smaller spatial extent. Despite the overall weaker dynamic intensity at 23.85 GHz, the wave-like character of the spin-wave modes is enhanced due to the effectively shorter wavelength (i.e. larger wave vector) associated with the higher frequency. This results in faster spatial phase oscillations and more pronounced propagating phase fronts within the geometrically constrained cap regions.

These animations provide direct visual evidence of the interplay between field angle, frequency, and structural geometry in shaping the collective behavior and spatial coherence of cap-localized spin-wave modes.

To gain deeper physical insight into the phase-resolved dynamics of cap-localized spin-wave modes described in Section 2.3, we performed additional micromagnetic simulations on a simplified linear chain of identical Ni nanotubes. Each nanotube was modeled with the same curvature, thickness, and dimensions as the tubes forming layers of the 3D woodpile structure. The goal of these simulations was to verify whether

the observed phase evolution among neighboring caps originates from dipolar coupling and to elucidate the underlying physical mechanism responsible for the phase flow.

S6.1 Dynamic magnetic potential in dependence of the field orientation

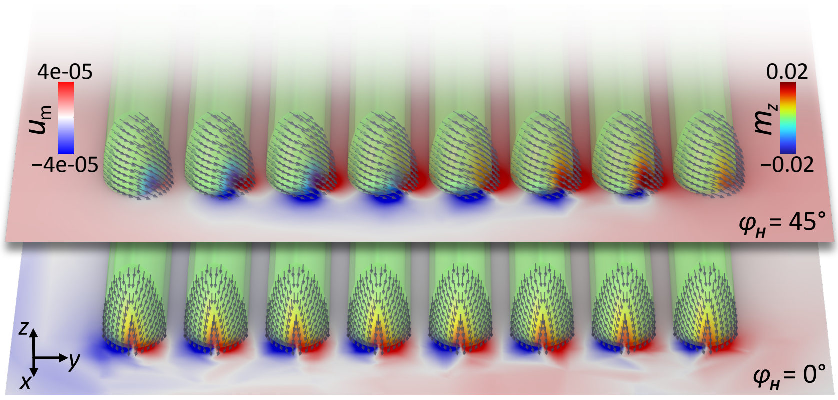

Figure S5 Simulated distributions of the dynamic magnetic scalar potential, u_m, around the curved caps for two representative orientations of the external magnetic field relative to the nanotube axis. All simulations were performed at $\mu_0 H = 0.77$ T and excitation frequency $f = 14.26$ GHz. The color within the nanotubes represents the out-of-plane component of the dynamic magnetization, m_z, while the arrows (visible only in the cap regions) indicate the static magnetization texture. The color maps of u_m illustrate alternating positive and negative potential regions $(+-+-)$ for the parallel field configuration, corresponding to a low-energy state of the dynamic dipolar field, and the distorted potential pattern that emerges when the field is rotated away from this orientation.

Figure S5 presents two representative configurations of the external magnetic field applied relative to the long axis of the nanotube array, together with the corresponding distributions of the dynamic magnetic scalar potential $u_\mathrm{m}(\mathbf{r}, t)$ around the curved caps.

When the field is aligned parallel to the nanotube axis, the dynamic potential alternates in sign between neighboring caps, forming a regular $(+-+-+-)$ sequence along the chain. This configuration minimizes the instantaneous dipolar energy associated with the dynamic magnetostatic fields, as the stray fields generated by adjacent caps partially compensate each other. In this configuration, the coupling between the caps is symmetric and energetically favorable, leading to stable, phase-coherent precession.

Upon rotating the external field away from this direction, the spatial pattern of u_m becomes distorted. Regions of equal sign in the potential start to overlap, producing zones of enhanced dynamic dipolar energy. The system therefore tends to reorient dynamically toward a configuration that restores phase alternation and thereby lowers the average dipolar coupling energy. This manifests as a spontaneous phase shift between neighboring caps. Such behavior provides a natural explanation for the observed phase flow in the woodpile structure: the system reduces the instantaneous dipolar field energy by adjusting the relative precessional phases of adjacent caps.

S6.2 Dependence on inter-cap spacing

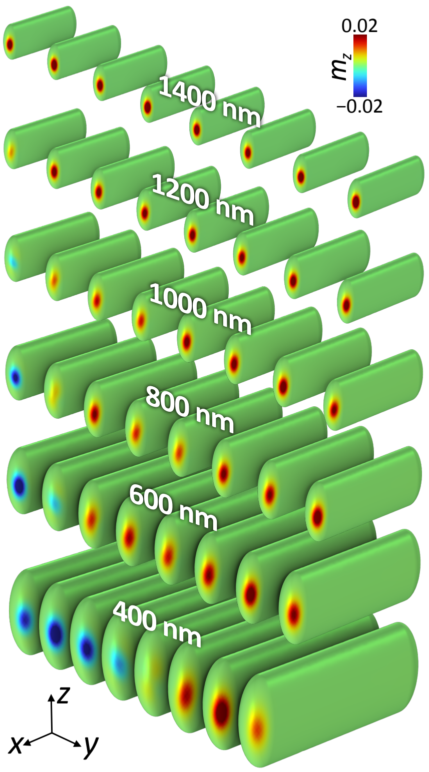

Figure S6 Dependence of the phase-resolved dynamics on the inter-cap spacing. Simulated linear chains of identical Ni nanotubes with varying center-to-center distance from 400 nm to 1400 nm (in 200 nm increments). All simulations were performed at an external magnetic field of $\mu_0 H = 0.77$ T, applied at an angle of 45° with respect to the tubes' long axis, and at an excitation frequency of $f = 14.26$ GHz. The color scale represents the out-of-plane component of the dynamic magnetization, m_z, highlighting the spatial phase change of the cap-localized spin-wave mode along each chain. With increasing separation, the phase correlation between neighboring caps gradually weakens and eventually vanishes, confirming the dipolar nature of the inter-cap coupling. All nanotubes share identical dimensions. Their relative sizes are visually rescaled in the figure solely for clarity and to accommodate all configurations within a single panel.

To further test the dipolar origin of the effect, we systematically varied the center-to-center distance between neighboring nanotubes within the same linear array. **Figure S6** summarizes the results for several separations. As expected for a dipolar interaction, the magnitude of the phase shift between caps decreases monotonically with increasing distance. For large separations, the dynamic fields of neighboring caps no longer overlap significantly, and the collective phase correlation disappears entirely. This trend confirms that the phase-resolved cap dynamics are not a direct consequence of local curvature or internal demagnetizing effects, but arise instead from the long-range dynamic dipolar coupling between the caps.

In summary, the toy-model simulations reveal that the phase coherence among cap-localized modes stems from the energetic balance of dynamic dipolar fields in neighboring curved caps. The configuration with alternating dynamic potential signs minimizes the magnetostatic energy, whereas misalignment of the field disrupts this balance and induces a compensating phase shift. The systematic decay of this effect with inter-cap spacing provides direct confirmation of its dipolar origin.
