# Supplementary figures and images for: Coherent Spin Waves in Curved Ferromagnetic Nanocaps of a 3D‐Printed Magnonic Crystal
Source: Small. 2025 Dec 17;22(7):e08983. doi: 10.1002/smll.202508983 (PMC12862454; doi:10.1002/smll.202508983)

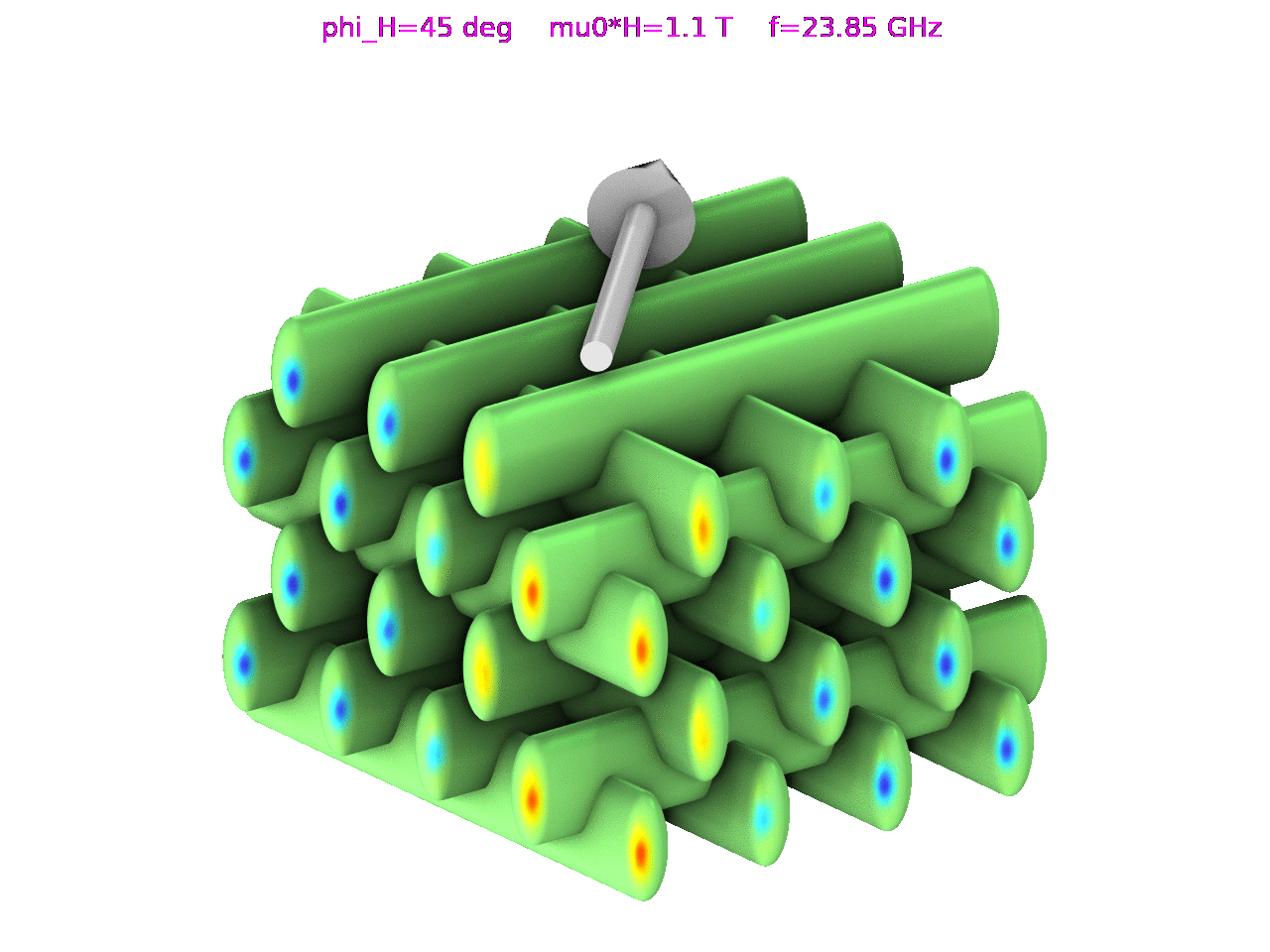

Supplement: Supplementary file 2 — Supporting Information [file SMLL-22-e08983-s001.zip › CapModes-24GHz-45deg-1_1T.gif]

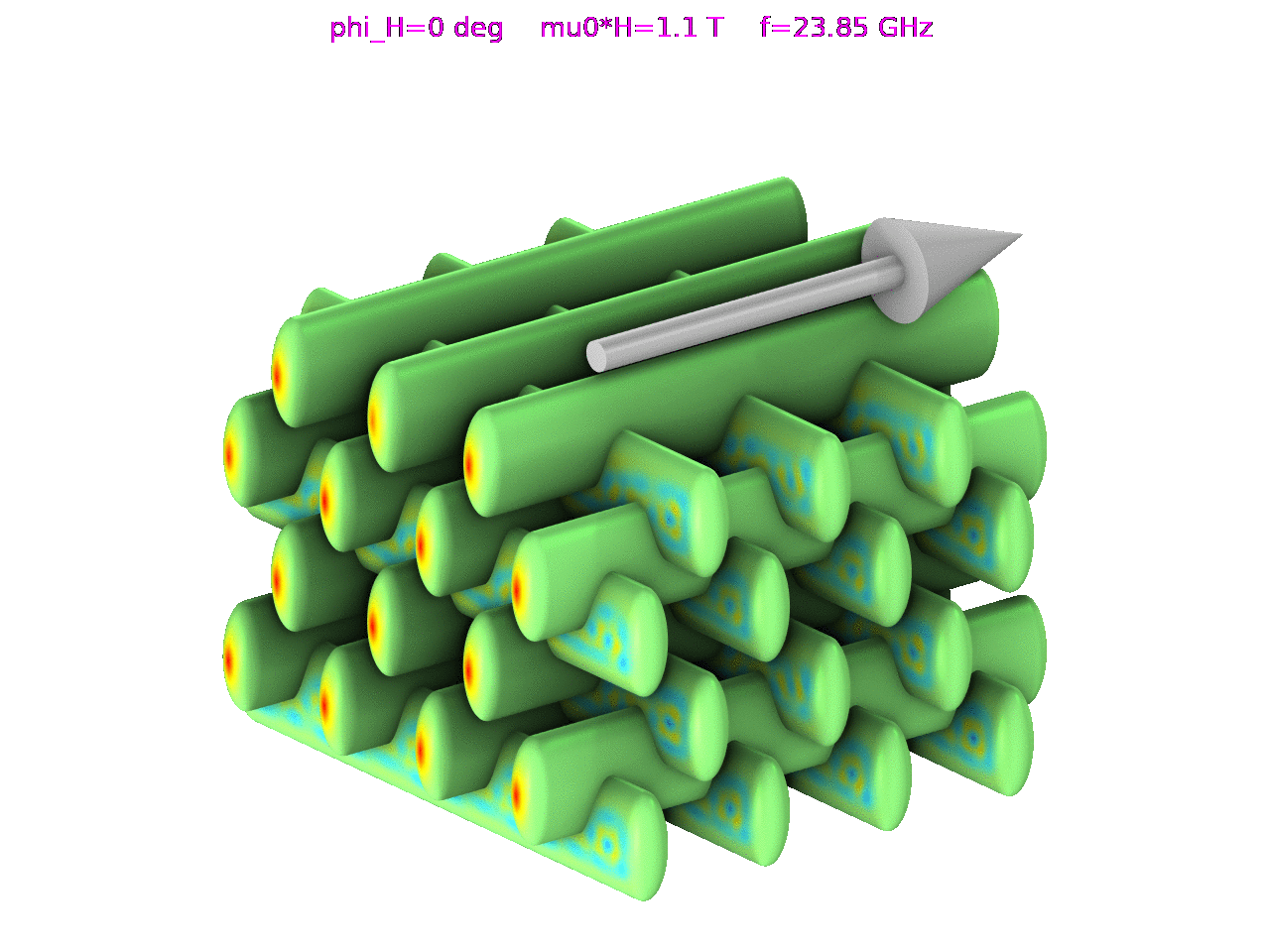

Supplement: Supplementary file 2 — Supporting Information [file SMLL-22-e08983-s001.zip › CapModes-24GHz-0deg-1_1T.gif]

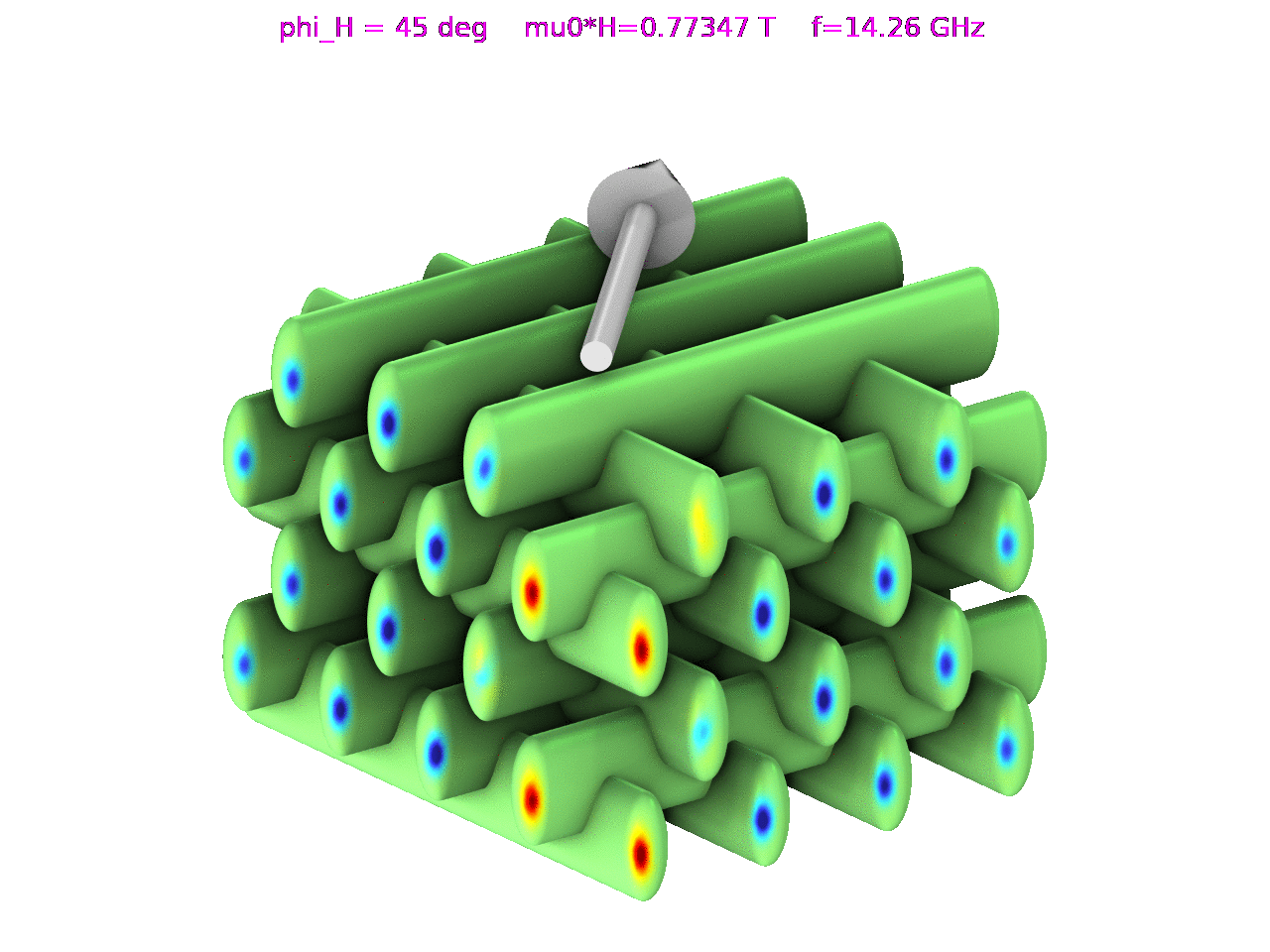

Supplement: Supplementary file 2 — Supporting Information [file SMLL-22-e08983-s001.zip › CapModes-14GHz-45deg-0_77T.gif]

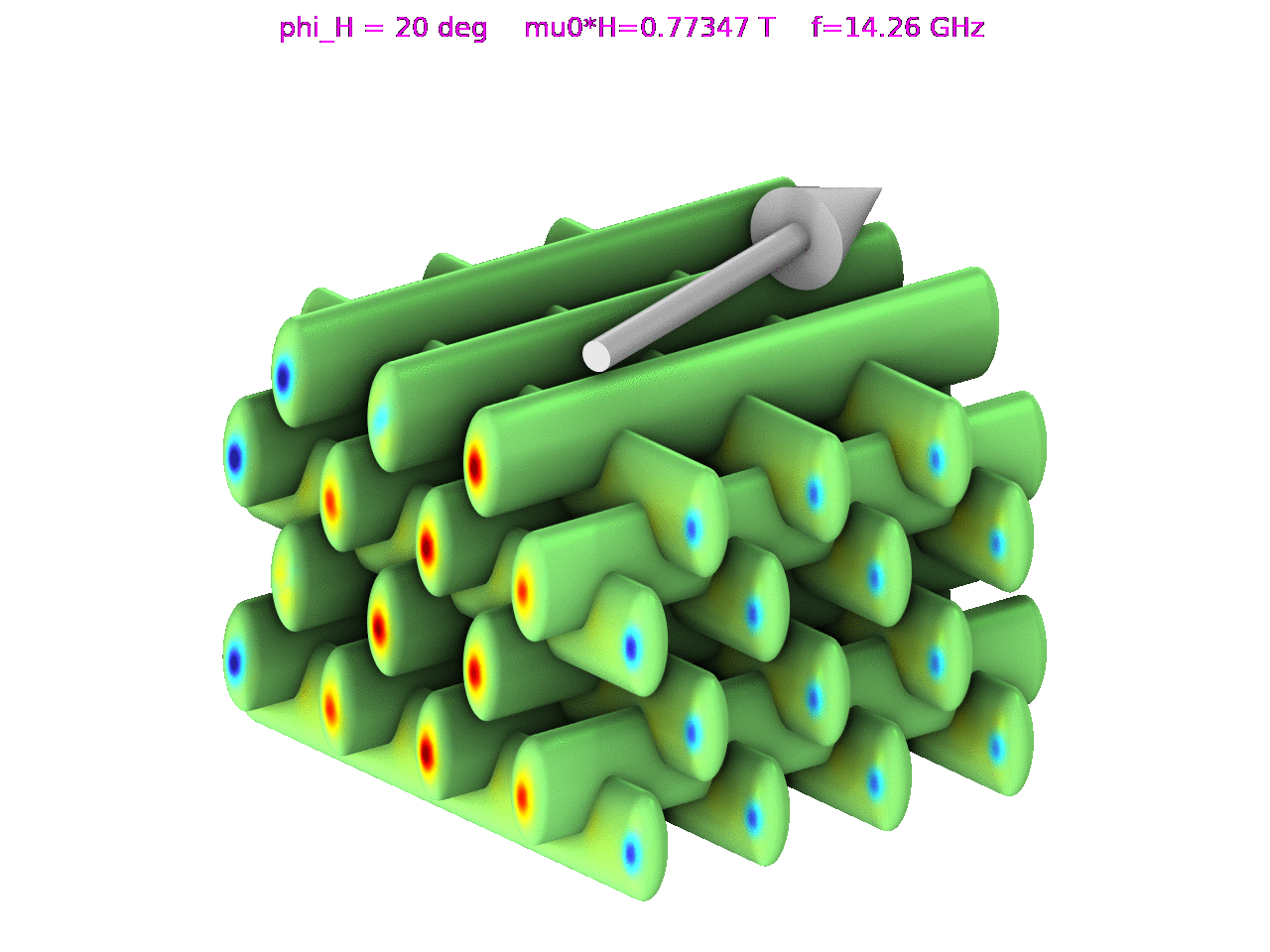

Supplement: Supplementary file 2 — Supporting Information [file SMLL-22-e08983-s001.zip › CapModes-14GHz-20deg-0_77T.gif]

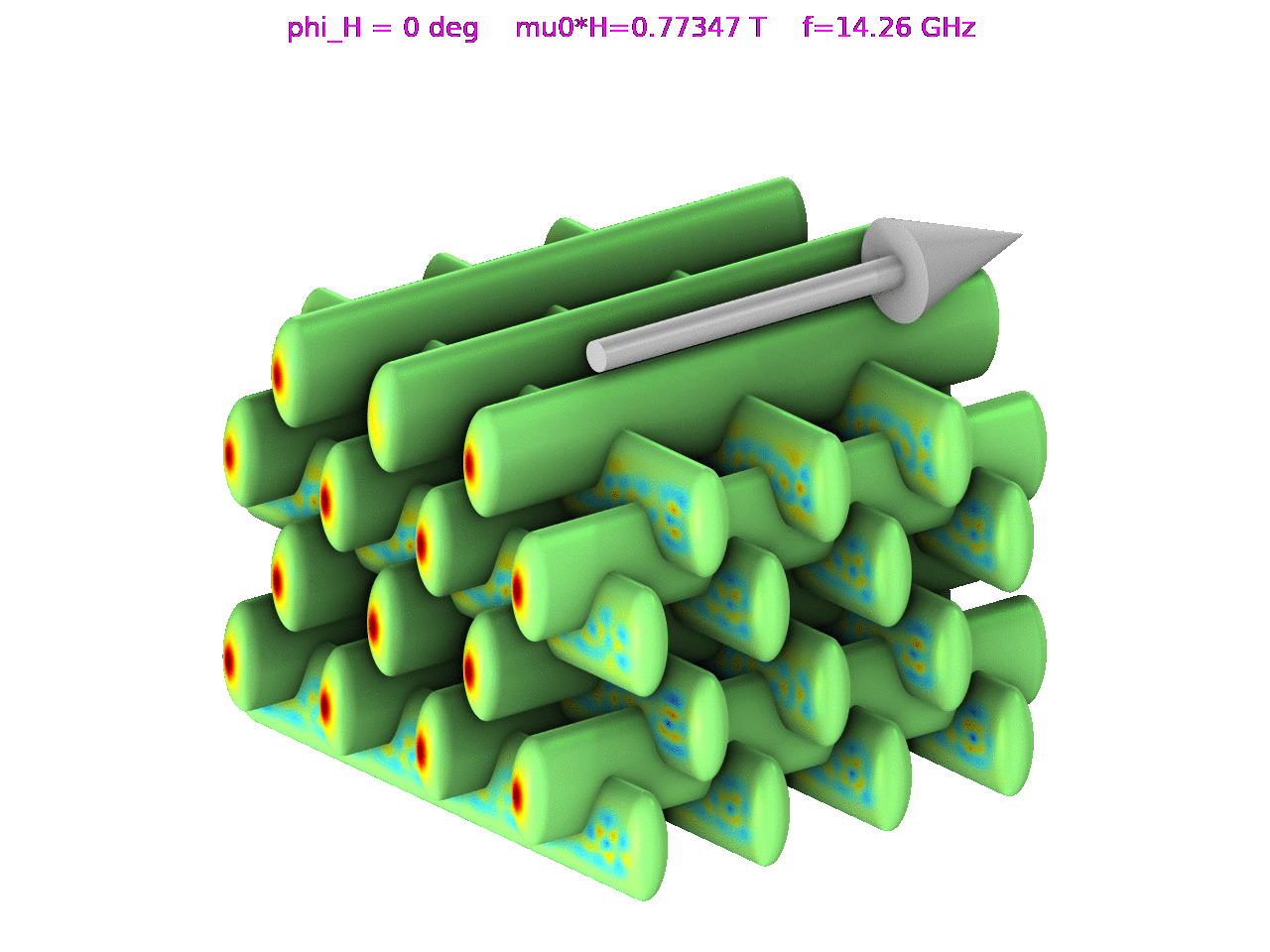

Supplement: Supplementary file 2 — Supporting Information [file SMLL-22-e08983-s001.zip › CapModes-14GHz-0deg-0_77T.gif]

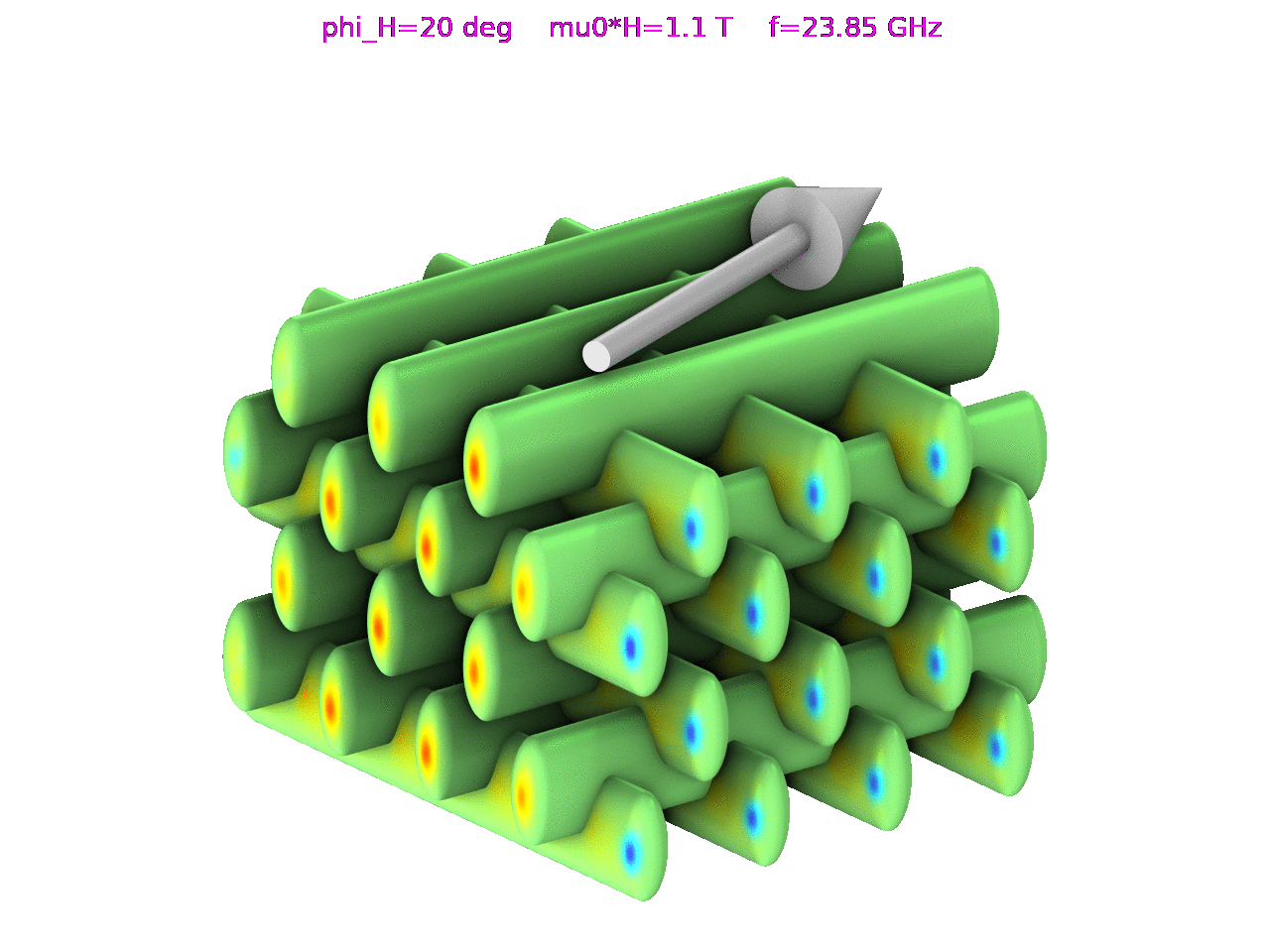

Supplement: Supplementary file 2 — Supporting Information [file SMLL-22-e08983-s001.zip › CapModes-24GHz-20deg-1_1T.gif]
